# Moisture-Related Shrinkage Behavior of Wood at Macroscale and Cellular Level

**DOI:** 10.3390/polym14225045

**Published:** 2022-11-21

**Authors:** Yufa Gao, Zongying Fu, Yongdong Zhou, Xin Gao, Fan Zhou, Huimin Cao

**Affiliations:** Key Lab of Wood Science and Technology of National Forestry and Grassland Administration, Research Institute of Wood Industry, Chinese Academy of Forestry, Beijing 100091, China

**Keywords:** wood drying, shrinkage behavior, macroscopic level, wood tracheid

## Abstract

Due to wood moisture sensitivity, shrinkage cracks tend to present wooden structures. These failures are caused by moisture-related shrinkage behavior. In order to avoid it, it is necessary to have a better understanding of shrinkage behavior. In this respect, studying the dimension changes in wood at different scales is of utmost significance for a better understanding of the shrinkage properties. Herein, the shrinkage behavior of Masson pines (*Pinus massoniana*) wood was investigated at macroscopic and cellular levels during moisture loss via digital image correlation using VIC-3D and digital microscopic systems, respectively. According to the full-field strain maps, shrinkage strain near the external face was higher than that at the internal face, which increased susceptibility to cracking at the external face of lumber. Additionally, the anisotropic shrinkage of wood was explored. The shrinkage ratio at the end of drying was about 5.5% in the tangential (T) direction and 3.5% in the radial (R) direction. However, at a cellular level, the shrinkage ratios in the T and R directions of earlywood tracheids were 7.13% and 2.46%, whereas the corresponding values for latewood tracheid were 9.27% and 5.52%, respectively. Furthermore, the maximum T/R shrinkage ratio at the macroscopic level (1.7) was found to be similar to the value of latewood tracheid (1.72). The earlywood showed high anisotropic, its T/R shrinkage ratio was 2.75. The macroscopic shrinkage was the result of the interaction of the tracheids of earlywood and latewood and was mainly dominated by latewood tracheids.

## 1. Introduction

Wood provides excellent performance, including superior strength-to-weight, heat insulation, and better seismic characteristics [1]. These advantages make it widely used in various applications, such as buildings, bridges, and furniture, etc. However, the sensitivity of wood moisture content (MC) to environmental conditions is the major stumbling block to being a safe and durable material. In a varying thermos-hygrometric environment, mechanical decay and dimensional changes may observe in the wood objects, especially in wooden heritage buildings [2]. The dimension can shrink as MC decreases, which can induce stresses and further lead to deformation and possibly failure [3,4]. Shrinkage cracks are present in most timber components, both in ancient and modern timber structures [2,5]. It is distressing that some valuable art works carried by wood, such as panel paintings, have been also damaged due to the properties of moisture-related behavior [6]. Therefore, a better understanding of wood moisture-related shrinkage behavior is crucial to ensure the long-term safety and reliability of the timber components.

The moisture-related dimensional variation, e.g., transverse anisotropy in tangential (T) and radial (R) directions, has been garnering considerable attention of researchers for several decades [7,8,9,10]. Traditionally, a ruler or micrometer is used to measure dimensional changes in wood during its moisture loss. However, providing an overall value through a relatively large surface area does not possess sufficient resolution for local assessment of shrinkage. Furthermore, the large-sized specimens may be distorted during drying, causing measurement errors [11]. In contrast, digital image correlation (DIC) is a non-contact full-field measurement technique that gathers accurate information about any displacements and strain in wood surface [12,13,14,15]. Kang et al. [16] tracked and measured the deformation of wood under simulated kiln drying conditions using an optical measurement system. Peng et al. [11,17] evaluated shrinkage in three anatomical directions from pith to bark, showing the potential of DIC technology for localized shrinkage analysis. Fu et al. [18] determined the shrinkage strain over each growth ring and visualized the full-field displacement and shrinkage strain distributions of wood at different moisture contents (MC). At the same time, DIC technology allows shrinkage behavior to be visualized and quantified, which is of great importance for understanding the generation of failures.

Macroscopic shrinkage of wood involves many microscopic level factors such as multilayered cell walls, cell shape, and earlywood–latewood alternation [19]. During moisture loss in a dry environment, the cell walls undergo shrinkage, which afterward spreads to the bulk of the wood. In this respect, the adequate interpretation of microscopic level deformations may contribute to the understanding of the shrinkage behavior of wood at the macro-level. Perré and Huber [20] evaluated the shrinkage of Douglas-fir (*Pseudotsuga menziesii*) and spruce (*Picea abies*) in the tissue using optical microscopy. Briefly, the early- and latewoods were isolated from the rest of the annual rings with the aid of a diamond wire saw, and the images of interesting zones at different MCs were grabbed to determine the relative shrinkage therein. Taguchi et al. [21,22] monitored the deformation behavior of tracheids during moisture uptake in yezomatsu (*Picea jezoensis*) wood at a cellular level via confocal laser scanning microscopy (CLSM). Almeida et al. [19] utilized environmental scanning electron microscopy (ESEM) to observe changes in cellular morphology and cell wall thickness, associated with tracheid shrinkage behavior of earlywood and latewood. These works provided useful insights into moisture-related shrinkage behavior of wood at the cellular level. Moreover, the interdependence of shrinkage behavior between macroscopic and cellular scales is poorly known and needs a thorough study [23].

The current research aims to elucidate the moisture-related shrinkage behavior of wood. The work is structured as follows: the specimen preparation and experimental methods is described in Section 2. Section 3.1 is focusing on the shrinkage behavior at the macroscopic level using a VIC-3D system. The dimensional changes in the tracheid (early- and latewood) during moisture loss is assessed at a cellular level with a digital microscopic system in Section 3.2. Special attention was paid to the interdependence of shrinkage between the macroscopic and cellular scales. Finally, the main conclusions are highlighted in Section 4.

## 2. Materials and Methods

### 2.1. Specimen Preparation

**Figure 1:**
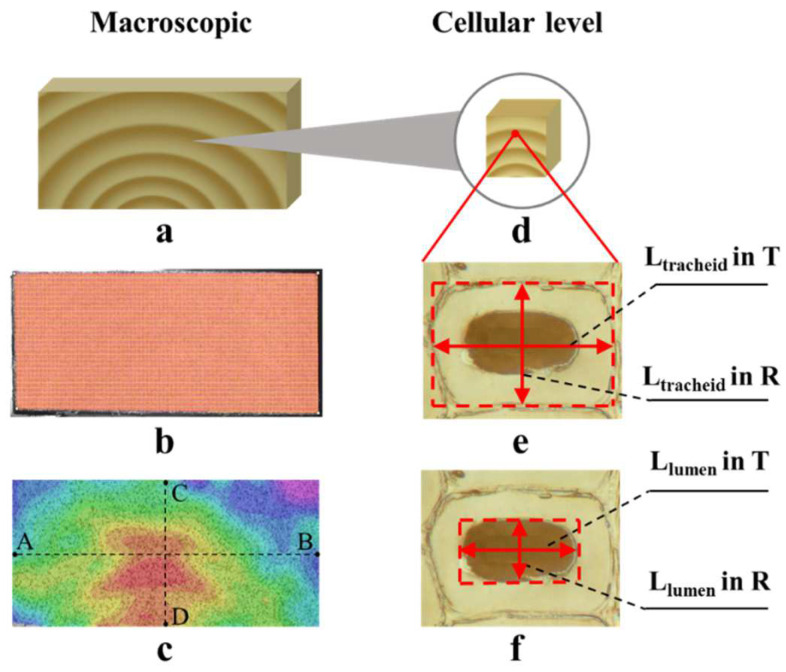
Schematic diagram of sample preparation for macroscopic (**a**) and cellular level (**d**), selected areas of interest (**b**), shrinkage strain analysis (**c**), determination for tracheid dimension (**e**), and determination for lumen dimension (**f**). The points A, B, C and D were selected to track the change in specimen dimension. L_tracheid_ in the T and R indicate the length of the tracheid in the tangential and radial directions, respectively. L_lumen_ in the T and R indicate the length of the lumen in the tangential and radial directions, respectively.

Masson pine (*Pinus massoniana*) is one of the most important and valuable plantation species in China, Masson pine is frequently used for a variety of applications in south part of China. Twenty-five-year-old Masson pine logs were collected from Paiyang forest farm (Chongzuo, China), and their basic density was 0.43 g/cm^3^. The logs were sawed into flatsawn lumbers with the dimension of 1500 mm × 120 mm × 50 mm (longitudinal (L) × T × R), then stored in a freezer at −4 °C to maintain the wood in green condition. One lumber was chosen with great care to avoid reaction wood and defects. Ten specimens with dimensions of 125 mm × 50 mm × 5 mm (T × R × L) were cut from the lumber. Select five of those specimens to realize the measurements at the macroscopic level, as shown in Figure 1. Cut the remaining five specimens in the dimensions of 5 mm × 5 mm × 5 mm (T × R × L) to measure the dimensional variation of early- and latewood at a cellular level; at least one whole growth ring in the sample should be assured.

### 2.2. Macroscopic Shrinkage Measurements

The shrinkage displacement and strain of specimens during drying were studied via digital image correlation using a VIC-3D system (Correlated Solutions, Inc., Irmo, SC, USA). The strain patterns were captured by two high-speed cameras set in front of the test specimen at different angles and distances. In order to enhance the measurement accuracy, the transverse surface was spray-painted to create a random black speckle pattern. The light-emitting diode (LED) was properly positioned to maintain the light environment during the experiment. The full-field displacements and strains in unstrained and deformed states were evaluated by comparing the positions of speckle pattern subsets in the consecutive images with respect to the DIC principle using analytical software [11,16]. The area of interest (AOI) from the cross section of wood (end surface) was chosen according to Figure 1b and divided into evenly spaced virtual grids (subsets). The parameters of the measuring setup were obtained conforming to a calibration procedure [15]. 

The specimens were dried at 50 °C in an oven (DKN611, Yamato, Japan). Different images of the end surface of specimen were selected using two cameras at pre-set interval of 1 min during drying process, which allowed one to minimize the errors caused by pseudo-in-plane strain from specimen distortion [17]. Rigid movement of the specimen, such as translation or rotation, would have exerted no effect on the displacement or strain calculation. As shown in Figure 1c, four points, referred to as A, B, C, D, were selected on the end surface of the specimen. The C–D line was drawn perpendicular to the annual rings to track the evolution of shrinkage strains from the internal face toward the external face of the wood sample. The distance between the two points (A, B) was calculated to determine the T dimension using VIC-3D analytical software. The distance between C and D points represented the R dimension of the specimen. The shrinkage ratios in the T and R directions (T_S_ and R_S_, respectively) were estimated from the formulae below:

(1) $$T_{S}=\frac{L_{\mathrm{Tg}}-L_{\mathrm{Td}}}{L_{\mathrm{Tg}}}\times 100\%$$

(2) $$R_{S}=\frac{L_{\mathrm{Rg}}-L_{\mathrm{Rd}}}{L_{\mathrm{Rg}}}\times 100\%$$

where L_Tg_ and L_Rg_ refer to the T and R dimensions of greenwood, and L_Td_ and L_Rd_ denote the T and R dimensions at the target MC. 

**Figure 2:**
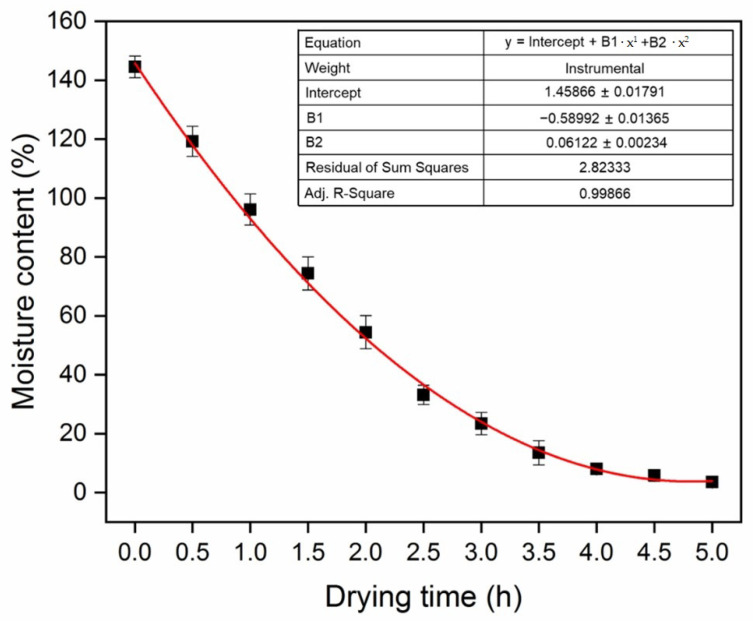
The fitting curve of MC as a function of drying time.

The actual MC of the samples could not be directly monitored during moisture loss. Therefore, the MC during drying was predicted from a series of empirical curves fitted to data acquired on the reference specimens under the same drying conditions, and the corresponding plots are given in Figure 2. In present work, MC of the specimens were calculated by weighing method. The specimens were dried at a constant temperature of 50 °C in a drying oven for 5 h and weighed every 30 min. Then, the oven-dried weight of the specimens was obtained after drying at 103 ± 2 °C for 24 h. Figure 2 displays the MC vs. drying time plot fitted using the regression equation below (Origin 2018 software):

MC = 1.46 − 0.59t + 0.61t^2^ (3)

### 2.3. Microscopic Observation

A digital microscopic system (VHX-6000, KEYENCE, Osaka, Japan) was used to collect strain patterns in wood cells. Prior to the measurements, the specimen cross-section was smoothened with a sliding microtome (RM2245, Leica, Wetzlar, Germany). The initial cell morphology image was microscopically observed in green condition. Then, the specimen was placed in the oven, at 50 °C, which was the same as macroscopic shrinkage measurements. During drying, the images were collected every 5 min for each specimen. At the same time, the weight was measured until remained constant. The images taken at 800× magnification were used for cell deformation assessment in early- and latewood specimens. Subsequently, the images were processed using Imaging J software to measure the diameters of tracheids (Figure 1). For the traced cells, the shrinkage ratio was obtained by comparing the dimensional changes in each tracheid at different MC levels. The shrinkage ratios in different directions were estimated as follows:

(4) $$T_{\mathrm{CS}}=\frac{L_{\mathrm{CTg}}-{\;L}_{\mathrm{CTd}}}{L_{\mathrm{CTg}}}\times 100\%$$

(5) $$R_{\mathrm{CS}}=\frac{L_{\mathrm{CRg}}-L_{\mathrm{CRd}}}{L_{\mathrm{CRg}}}\times 100\%$$

where T_CS_ represents the T shrinkage ratio of tracheid or lumen, L_CTg_ denotes the T dimension of tracheid or lumen in green condition, and L_CTd_ refers to the T dimension of tracheid or lumen at the target MC. Additionally, R_CS_ represents the R shrinkage ratio of tracheid or lumen, L_CRg_ corresponds to the R dimension of tracheid or lumen in green condition, and L_CRd_ refers to the R dimension of tracheid or lumen at target MC.

## 3. Results

### 3.1. Shrinkage Behavior at Macroscopic Level

#### 3.1.1. Full-Field Distribution of Shrinkage Strain

**Figure 3:**
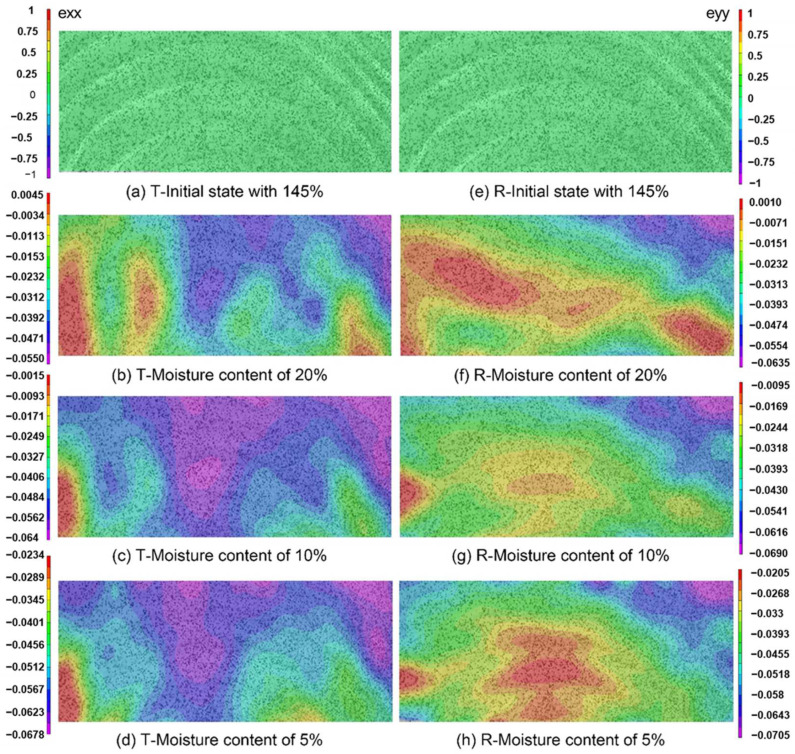
The distribution of tangential (**a**–**d**) and radial (**e**–**h**) shrinkage strains at different MC, where exx and eyy denote the strain in tangential and radial directions, respectively. The color bars indicate the amount of shrinkage strain.

The color map of full-field displacement is based on the DIC principle, which ensures that the displacement in specimen surface is available for visual inspection and quantitative analysis [14]. The full-field distributions of shrinkage strain in the T and R directions at different moisture contents are shown in Figure 3. Herein, the principal strains exx and eyy correspond to the deformations in the T and R directions, respectively, and the negative and positive values represent the proportions of wood contraction (shrinkage strain) and expansion (expansion strain). Despite multiple repetitions, the most representative full-field shrinkage strain was selected to analyze shrinkage behavior. The color map clearly visualizes the progress of strain gradient in the specimen R × T surface during moisture loss. Figure 3a–d sketch the strain distributions in the T direction for several MC levels. According to these results, the T shrinkage strain in the middle exceeded those on the left or right sides of the specimen, which was explained by the fact that the annual rings orientation in the middle position was parallel to the T direction. The T shrinkage strain at the middle part of the specimen was equal to the total shrinkage strain. However, on the side regions, the annual rings orientation was inclined with respect to the T direction, and the T shrinkage strain was a horizontal component of the total shrinkage strain. In addition, the shrinkage strain near the external face exceeded that at the internal face. This was owing to the larger radius of the annual ring near the external surface as well as the small angle between the former and the T direction, resulting in the greater horizontal component of the total shrinkage strain. The shrinkage trend was consistent with the upward warping into a trough-like shape of flatsawn lumber during drying [24,25]. The stress concentration at the specimen boundary near the external face might led to the occurrence of cracks. With the decrease in MC, the specimen surface shrank and reached the maximum strain at the MC of 5%. The shrinkage strain distribution in the R direction is shown in Figure 3e–h. The shrinkage strain on the left and right sides of the specimen was higher than that in the middle. This could be justified by the normal orientation of the annual rings in the middle position and their parallel orientation on the side regions relative to the R direction, yielding the larger R shrinkage strain at sides zones due to the horizontal component of the T strain of annual rings. In addition, the R shrinkage strain was consistent with the ring shape. As seen in Figure 3, the colors corresponding to drying-induced shrinkage strains were alternately changing similar to those of the growth ring, which was due to the different shrinkage strains of early- and latewood within each growth ring [16,26]. 

**Figure 4:**
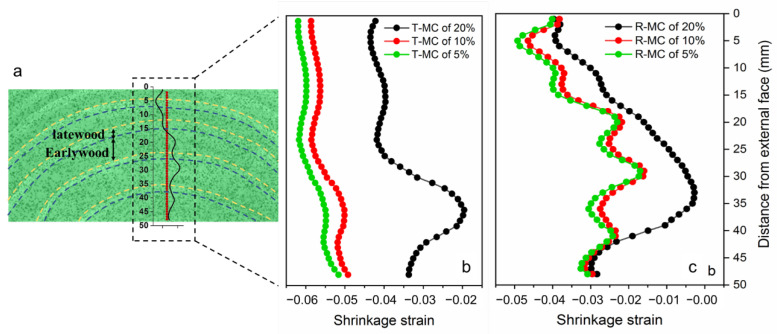
(**a**) Tangential shrinkage strain distribution diagram at initial state and the growth rings are distinguished by yellow and blue dashed lines. The dashed part shows the selection of the test points and the variation shrinkage strains with distance. The variation of tangential (**b**) and radial (**c**) shrinkage strains from the external to the internal face at different MC values.

The shrinkage strain variation patterns from the internal to the external face at different moisture contents are given in Figure 4. The alternating wave patterns of low and high values corresponding to the T and R shrinkage strains were attributed to significant differences in early- and latewood within each growth ring [11,16,17]. As expected, the T and R shrinkage strain changed like an alternating wave. The strain variation in the R direction was more consistent with the distribution of the growth ring than that in the T direction. This result was explained in terms of the combined effect of the interactions between early- and latewood. The shrinkage between latewood and earlywood was not affected by each other in the R direction. However, in the T direction, the strong bands of latewood forced the weak bands of earlywood to change to about the same extent as the latewood [5]. Moreover, it was clear that the shrinkage strain demonstrated an increasing tendency both in the T and R directions with the MC decreasing. The maximum T shrinkage strain values for the MC of 20%, 10%, and 5% were approximately −0.04, −0.055, and −0.06, correspondingly. In the R direction, the maximum strain values were about −0.03, −0.045, and −0.05 at the MC of 20%, 10%, and 5%, correspondingly.

#### 3.1.2. Wood Anisotropic Shrinkage

**Figure 5:**
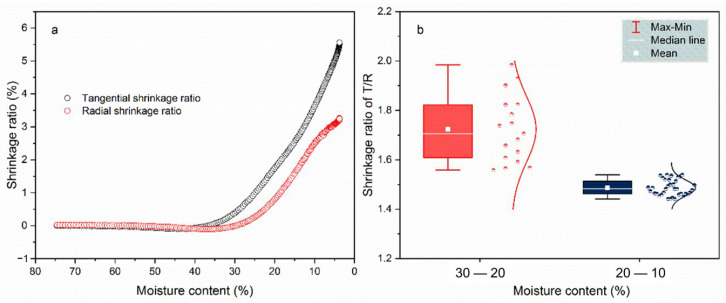
The shrinkage ratio (**a**) in T and R directions and T/R ratio (**b**) at different MC regions.

Wood is an anisotropic material, and there is a clear difference between tangential and radial shrinkages. The ratio of shrinkage in the T and R directions varied with MC, as shown in Figure 5a. The slight shrinkage occurred below 35%, which was associated with fiber saturation point (FSP). The FSP was the MC at which the cell walls are fully saturated with bound water and with no free water in the lumen [27]. Below the FSP, the dimension of wood changes as bound water was lost from the cell wall [28,29]. When the MC was below 25%, the shrinkage ratio of the specimen exhibited a linear decrease in the T and R directions. At the end of drying, the shrinkage ratio was about 5.5% in the T direction and 3.5% in the R direction. 

Figure 5b shows the T/R ratio of two MC regions. Since the shrinkage was small with the MC beyond 30%, the T/R ratio was plotted with the MC below 30%. The boxplot and scatterplot distributions of the T/R ratio indicate that the specimen was more anisotropic in the MC region of 30–20%. The higher anisotropic shrinkage resulted in the larger drying stress. This conclusion is consistent with the fact that the small cracks occur during the initial drying of the specimen surface [30]. The average value of T/R was about 1.7 and 1.5 at the MC regions of 30–20% and 20–10%. This was close to the commonly accepted results, the T shrinkage is assumed twice as large as the radial shrinkage [31].

### 3.2. Shrinkage Behavior at Cellular Level

#### 3.2.1. Shrinkage Ratio of Tracheid and Lumen

**Figure 6:**
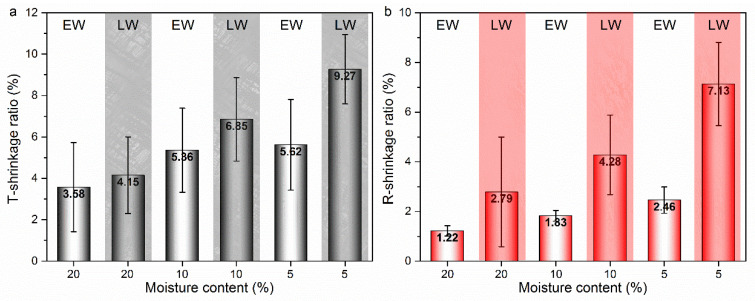
The shrinkage ratio of tracheid in early- and latewood in tangential (**a**) and radial (**b**) directions. White background for earlywood, red and black background for latewood.

The shrinkage properties of wood at the macroscopic level are dictated by its complex anatomical structures. Studying dimensional changes at a cellular level may contribute to a better understanding of shrinking anisotropy. Figure 6 displays the shrinkage ratio of tracheid of early- and latewood. As expected, shrinkage was pronounced in the latewood cells rather than in earlywood, both in the T and R directions [19,21,22]. The shrinkage ratio of the tracheid in early- and latewood exhibited a similar trend, both increasing with MC decreasing. At the end of drying, the earlywood tracheid shrinkage ratios in the T and R directions were 5.52% and 2.46%, respectively. The shrinkage ratios of latewood tracheid in the T and R directions were 9.27% and 7.13%, respectively. Significant differences existed in the wood shrinkage between macroscopic and cellular levels. 

In the T direction, the earlywood tracheid shrinkage ratio was similar to that of the bulk material. In contrast, the shrinkage ratio of latewood tracheid was greater than that at the macroscopic scale. The arrangements of various tissues and cell types were related to this difference. The early- and latewood cells were arrayed in parallel and series in the T and R directions, respectively. In this respect, the T shrinkage was affected by a combination of early- and latewood tracheids, which were mutually constrained by each other. According to Figure 6, the tracheid shrinkage of the latewood was greater than that of the earlywood. Therefore, the strong tangential bands of latewood forced the weak bands of the earlywood to shrink in the tangential direction to about the same extent as in the latewood. There was also some decrease in the latewood shrinkage because of the resistance of the earlywood. Macroscopic shrinkage seems to be dominated by the latewood tracheids in the T direction, but constrained by the earlywood tracheids. The similar results were found by Patera et al. [32]. The swelling and shrinkage behavior of single earlywood, single latewood and earlywood/latewood combined have been reported. The swelling ratio is higher in latewood and lower in earlywood. For the combined region, its swelling ratio was between the early- and latewood, which indicates the mutual restraining effect of the early- and latewood.

In the R direction, shrinkage was the average among the independent early- and latewood tracheids [33,34]. In this study, the macroscopic shrinkage ratio (3.5) is less than the average (4.79) of the early- and latewood shrinkage. The ray restraint theory can explain this result very well. The radially oriented ray cells have a minimal longitudinal shrinkage, and it is presumed to be stronger than surrounding tissue, thus restraining the macroscopic radial shrinkage [35,36,37]. Furthermore, this was also owing to a decrease in the radial shrinkage of earlywood tracheids while attached to the latewood. The earlywood was afterwards forced to drastically shrink in the same tangential direction as the latewood, undergoing the decrease in shrinkage in the R dimension through the Poisson effect [5]. The shrinkage behavior at the macroscopic level in the R direction was affected by both the early- and latewood tracheids, but not simply by their accumulation.

**Figure 7:**
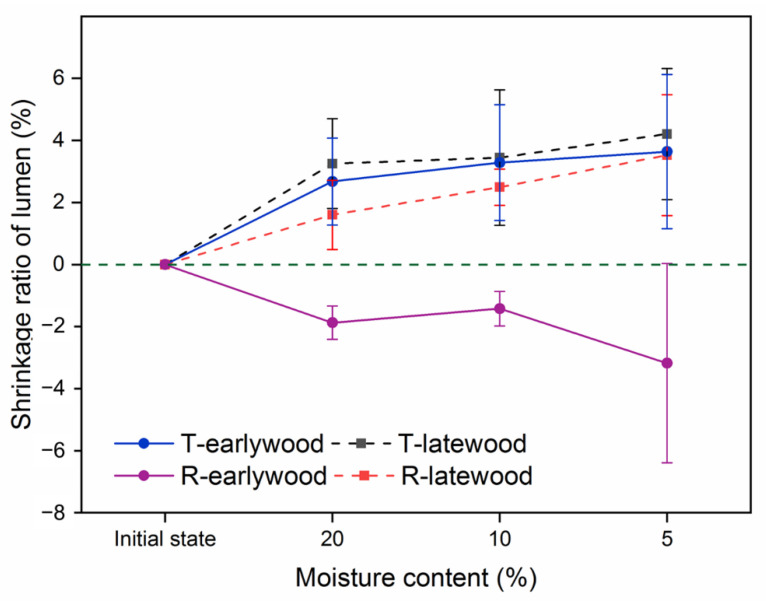
The shrinkage ratio of the cell lumen in the tangential and radial directions of early- and latewood.

The relevant changes in T and R directions in lumen versus MC are illustrated in Figure 7. Noticeable shrinkage was observed in both the T and R directions of the lumen with the moisture loss. Interestingly, the earlywood lumen in the R direction exhibited a significant expansion, whereas the T diameter shrank with the loss of moisture. This might also be due to the Poisson effect of the tracheid mentioned above [5,22]. It is noteworthy that nearly all pits were on the radial walls and seldom located on the tangential walls of longitudinal tracheids. Thus, the shrinkage of the tangential walls was greater than that of the radial walls. The rapid shrinkage in the T direction of the tracheid during moisture loss squeezed the dimensions in the R direction, resulting in the lumen swelling in the same direction.

#### 3.2.2. Shrinkage Ratio of Tracheid and Lumen

**Figure 8:**
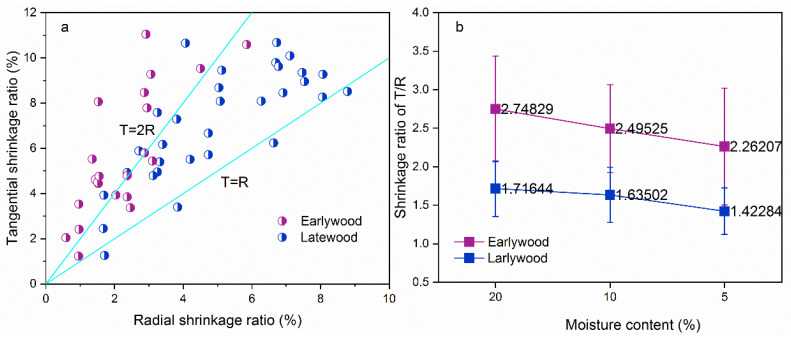
Anisotropic shrinkage between the T and R directions at the cellular level: (**a**) T shrinkage ratio vs. R ratio of early- and latewood tracheids and (**b**) T/R ratio of early- and latewood tracheids at different MCs.

Figure 8 shows the differential shrinkage of early- and latewood tracheids between the T and R directions. The T shrinkage as a function of R shrinkage was plotted in Figure 8a. The latewood tracheid shrinkage ratio was distributed between the T = R and T = 2R. In contrast, the shrinkage ratio was around the line of T = 2R for earlywood tracheid, which meant high anisotropic shrinkage. The T/R shrinkage ratio of early- and latewood tracheids is demonstrated in Figure 8b. The earlywood tracheids T/R shrinkage ratio (from 2.26 to 2.74) was greater than that of the latewood tracheids (from 1.42 to 1.71). The maximum T/R values of early- and latewood were 2.74 and 1.71, as the MC was 20%. The difference in cell wall thickness between early- and latewood is an essential factor in this phenomenon. Patera et al. [38] investigated the swelling behavior of four different wood tissues and found the dependence of anisotropy on the porosity. The anisotropy may derive from the stronger existence (in proportion) of the constraint effect of the S1 and S3 layers. Therefore, the earlywood shows very thin cell walls (S2) with the high porosity, and present higher anisotropic characteristics. Furthermore, the deformation of earlywood cells in the R direction were strongly restraining by wood rays. The reduction effect of wood rays in latewood may be explained by the thicker and more dense structure [32,39]. 

## 4. Conclusions

This work provided the basic deformation data at two scales that contributes to a deeper understanding of wood shrinkage behavior and necessary input in predictive mechanical models of wood drying. At the macroscopic scale, the shrinkage ratio in the T and R directions reached the maximum of 5.5% and 3.5%, respectively. For wood cells, the maximum T and R shrinkage the earlywood tracheids were 5.52% and 2.46% at the end of drying, and the shrinkage ratio of latewood tracheids were 9.72% and 7.13, respectively. Regarding the anisotropic shrinkage, the most obvious shrinkage anisotropy was presented in MC range of 30–20%, where the average T/R ratio was 1.7. At a cellular level, the T/R shrinkage ratios of the earlywood tracheids were between 2.26 to 2.74, whereas those of the latewood tracheids ranged from 1.42 to 1.72. There were significant differences between the microscopic and macroscopic both in shrinkage ratio and anisotropy behavior. The interaction between early- and latewood and the inhibitory effect of the ray tissue are the reasons for this result. Therefore, the interdependence of shrinkage between macroscopic and cellular level should be investigated from the arrangements of various tissues, cell types and cell wall structures in the future perspectives.

## Data Availability

The data presented in this study are available upon request from the corresponding author.
